# Comparative Evaluation of Color Stability and Surface Roughness of Bulk-Fill and Nanohybrid Composites Following Long-Term Mouthrinse Exposure: An In Vitro Study

**DOI:** 10.7759/cureus.84320

**Published:** 2025-05-18

**Authors:** Shivani Bansal, Saurabh Gupta, Saru Gupta, Poonam Bogra, Rajinder Bansal, Vishakha Grover, Seema Gupta

**Affiliations:** 1 Conservative Dentistry and Endodontics, J.N. Kapoor D.A.V. Centenary Dental College, Yamunanagar, IND; 2 Conservative Dentistry and Endodontics, All India Institute of Medical Sciences, Jammu, IND; 3 Pediatric and Preventive Dentistry, Maharishi Markandeshwar College of Dental Sciences and Research, Ambala, IND; 4 Conservative Dentistry and Endodontics, Guru Nanak Dev Dental College and Research institute, Sunam, IND; 5 Periodontology, Dr. Harvansh Singh Judge Institute of Dental Sciences and Hospital, Chandigarh, IND; 6 Orthodontics, Kothiwal Dental College and Research Centre, Moradabad, IND

**Keywords:** color, composite resin, mouthwash, nanocomposite, stability, surface roughness

## Abstract

Introduction: Composite resin restorations are widely used in restorative dentistry; however, their long-term aesthetic success depends on color stability and surface integrity. Daily exposure to mouthwashes may influence these properties. This study aimed to evaluate and compare the effects of different mouthwash formulations on the color stability and surface roughness of Tetric N-Ceram Nanohybrid (Ivoclar Vivadent, Schaan, Liechtenstein) and Tetric N-Ceram Bulk-Fill (, Ivoclar Vivadent, Schaan, Liechtenstein) composites.

Materials and Methods: 80 disc-shaped specimens (n = 40/group) of nanohybrid (Tetric N-Ceram), and bulk-fill composite (Tetric N-Ceram) were fabricated and polished according to the manufacturer’s instructions. Each group was further divided into four subgroups (n = 10): control (distilled water), Listerine Cool Mint® (alcohol-based mouthwash, Johnson & Johnson, New Brunswick, USA), Phos-Flur® (fluoride-based mouthwash, Colgate-Palmolive, New York, USA), and Rexidine® (0.2% chlorhexidine-based mouthwash, Indoco Remedies Ltd., Mumbai, India). Specimens were immersed in 30 mL of the respective solution for five min per cycle, three times daily, for 60 days, simulating one year of clinical exposure. The color change (ΔE) was measured using a VITA Easyshade® V spectrophotometer (VITA Zahnfabrik, Bad Säckingen, Germany), and the surface roughness (Ra) was assessed using a contact profilometer (Zeiss, Oberkochen, Germany). Scanning electron microscopy (SEM) (Zeiss, Oberkochen, Germany) analysis with 3D surface plots using ImageJ software (National Institutes of Health, Bethesda, Maryland, USA) provided a complementary morphological evaluation. Data were analyzed using one-way analysis of variance (ANOVA) and post-hoc Tukey's test (p < 0.05).

Results: Significant color changes were observed in both composites, with Listerine causing the highest ΔE values, particularly in the bulk-fill group. Rexidine caused notable discoloration, whereas Phos-Flur had minimal effects. The surface roughness increased significantly in both materials after exposure, with the nanohybrid composites showing greater sensitivity to surface alterations.

Conclusion: The mouthwash formulation significantly affected the aesthetic and surface characteristics of composite resins. Nanohybrid composites demonstrated better color stability, but were more susceptible to surface roughness changes than bulk-fill composites.

## Introduction

Composite resin restorations have become the cornerstone of modern restorative dentistry because of their aesthetic appeal, mechanical strength, and ability to mimic natural tooth structures. The clinical success and longevity of these restorations are heavily influenced by two critical properties: color stability and surface roughness [1,2]. Color stability ensures that restorations maintain their aesthetic integrity over time, aligning with the patient’s natural dentition and meeting aesthetic expectations, which is particularly crucial for anterior restorations where visibility is high [3]. On the other hand, surface roughness plays a vital role in plaque accumulation, gingival health, and wear resistance [4]. Smooth surfaces have been associated with reduced bacterial adhesion and improved biocompatibility [4]. Discoloration and surface degradation can compromise these properties, leading to restoration failure, patient dissatisfaction, and the need for expensive replacements [3,5]. Therefore, understanding the factors that influence color stability and surface roughness is essential for optimizing material selection and achieving favorable clinical outcomes.

The Tetric N-Ceram Nanohybrid (Ivoclar Vivadent, Schaan, Liechtenstein), a nanofilled composite resin, combines nano- and micro-sized filler particles to enhance the polishability and color retention. Its uniform filler distribution reduces light scattering, enabling superior aesthetic and mechanical performances [6]. In contrast, the Tetric N-Ceram Bulk-Fill (Ivoclar Vivadent, Schaan, Liechtenstein) was designed for placement in thicker increments, incorporating larger filler particles and a specialized photoinitiator system that facilitates deeper light penetration and curing [7]. Although this simplifies clinical procedures, it may result in differences in the surface texture and color stability, especially under conditions that simulate long-term oral exposure [8,9].

Mouthrinses are widely used in oral hygiene routines and subject restorations for various chemical and mechanical stresses [10]. Alcohol-based formulations can degrade the resin matrix through solvent action, leading to erosion and discoloration [10,11]. Fluoride-based mouthrinses, although protective for enamel, may alter filler-matrix interfaces, influencing surface texture over time [12]. Chlorhexidine-containing rinses, known for their antimicrobial efficacy, have also been associated with surface staining and microabrasion upon prolonged use [13]. Given their daily use by patients, these solutions may induce cumulative effects on restorative materials that are not fully captured by short-term or isolated evaluation [10].

Some prior studies have assessed the effects of mouthrinses on composite resins, primarily by examining color stability via spectrophotometry and surface roughness using profilometry [2,5,14]. However, few studies have integrated both parameters with advanced surface characterization [2,14]. This study addresses this gap by employing a novel, multi-assessment approach involving spectrophotometric analysis of color change, surface topographical analysis using a surface profilometer and three-dimensional (3D) surface plotting of scanning electron microscopy (SEM) images using ImageJ software. This comprehensive methodology enabled a more detailed evaluation of both aesthetic and microstructural changes in nanohybrid and bulk-fill composites following exposure to commonly used mouthrinses.

Therefore, the aim of this study was to evaluate and compare the effects of different commercially available mouthrinses on the color stability and surface roughness of two types of composite resins: Tetric N-Ceram Nanohybrid and Tetric N-Ceram Bulk-Fill. Specifically, this study sought to assess the extent of color change (ΔE) and change in surface roughness in both composites following immersion in three types of mouthrinse formulations: alcohol, fluoride, and chlorhexidine-based. The null hypothesis stated that no significant differences in color stability or surface roughness would occur between the Tetric N-Ceram Nanohybrid and Tetric N-Ceram Bulk-Fill composite resins after exposure to various mouthrinses.

## Materials and methods

Study design and setting

This in vitro experimental study was conducted at the Department of Conservative Dentistry and Endodontics, J.N. Kapoor D.A.V. Centenary Dental College, Yamunanagar, India, from July 2024 to October 2024. The study was conducted as a controlled, comparative, in vitro experiment under standardized laboratory conditions, and did not involve human participants, biological tissues, or animal subjects, and ethical approval was not required. This study was conducted in accordance with the principles of the Declaration of Helsinki.

Sample-size estimation

The sample size was calculated using G*Power software (version 3.1.9.3; Franz Faul, University of Kiel, Germany) based on the difference between two independent group means (t-test) with an effect size of 0.59, an alpha error probability of 0.05, and a power of 0.80. By applying a formula of n = (Z_1-__α/2_+Z_1-__β_)^2 ^* SD^2^ / effect size^2 ^(Z_α/2_=1.96, Z_β_=0.84 and pooled SD= 0.33), this yielded the required sample size of 36 specimens per group, which was increased to 40 to compensate for the potential material loss. Thus, 80 specimens (two materials * four exposure groups) were prepared and analyzed.

Inclusion and exclusion criteria

The inclusion criteria were freshly fabricated disc-shaped specimens of the Tetric N-Ceram Nanohybrid and Tetric N-Ceram Bulk-Fill composites with uniform dimensions of 8 mm in diameter and 4 mm in thickness, exhibiting complete polymerization and free of surface defects or air entrapments. Specimens were excluded if they exhibited any visible cracks, incomplete curing, surface irregularities identified through initial SEM analysis, or if they were compromised by contamination during preparation or storage.

Specimen preparation

80 specimens were fabricated (40 samples per group) by inserting the composite materials into Teflon molds with standardized dimensions (8 mm × 4 mm). Each material was condensed into a mold using a hand-held instrument. Excess material was removed by placing a transparent Mylar strip (SS White Co., Philadelphia, USA) and pressing it on a 1-mm-thick glass plate. Light curing was performed through a glass plate using a light-emitting diode (LED) curing unit (Coltene Spec 3 LED, Coltene, Korea) with a wavelength range of 430-490 nm for 20 seconds. A radiometer (Hilux Curing Light Meter, Benlioglu Dental Inc., Ankara, Turkey) was used to evaluate the luminous intensity of the curing light apparatus. After curing, all specimens were rinsed with distilled water and stored in artificial saliva at 37°C for 24 hours in an incubator (Dentsply Sirona, Charlotte, USA) to ensure complete polymerization.

After curing, the top surface of each specimen was finished and polished by a single experienced calibrated endodontist (Shivani Bansal) in accordance with the manufacturer’s instructions. Finishing and polishing were achieved using Super Snap polishing disks (Shofu Inc., Kyoto, Japan) mounted on a high-speed handpiece to ensure surface uniformity and minimize irregularities.

Grouping and immersion protocol

The two composite groups were divided into four subgroups (n = 10) based on the immersion solution: Subgroup 1, distilled water (control); Subgroup 2, Listerine Cool Mint® (alcohol-based mouthrinse, Johnson & Johnson, New Brunswick, USA); Subgroup 3, Phos-Flur® (fluoride-based mouthrinse, Colgate-Palmolive, New York, USA); and Subgroup 4, Rexidine® (0.2% chlorhexidine-based mouthrinse, Indoco Remedies Ltd., Mumbai, India).

**Figure 1:**
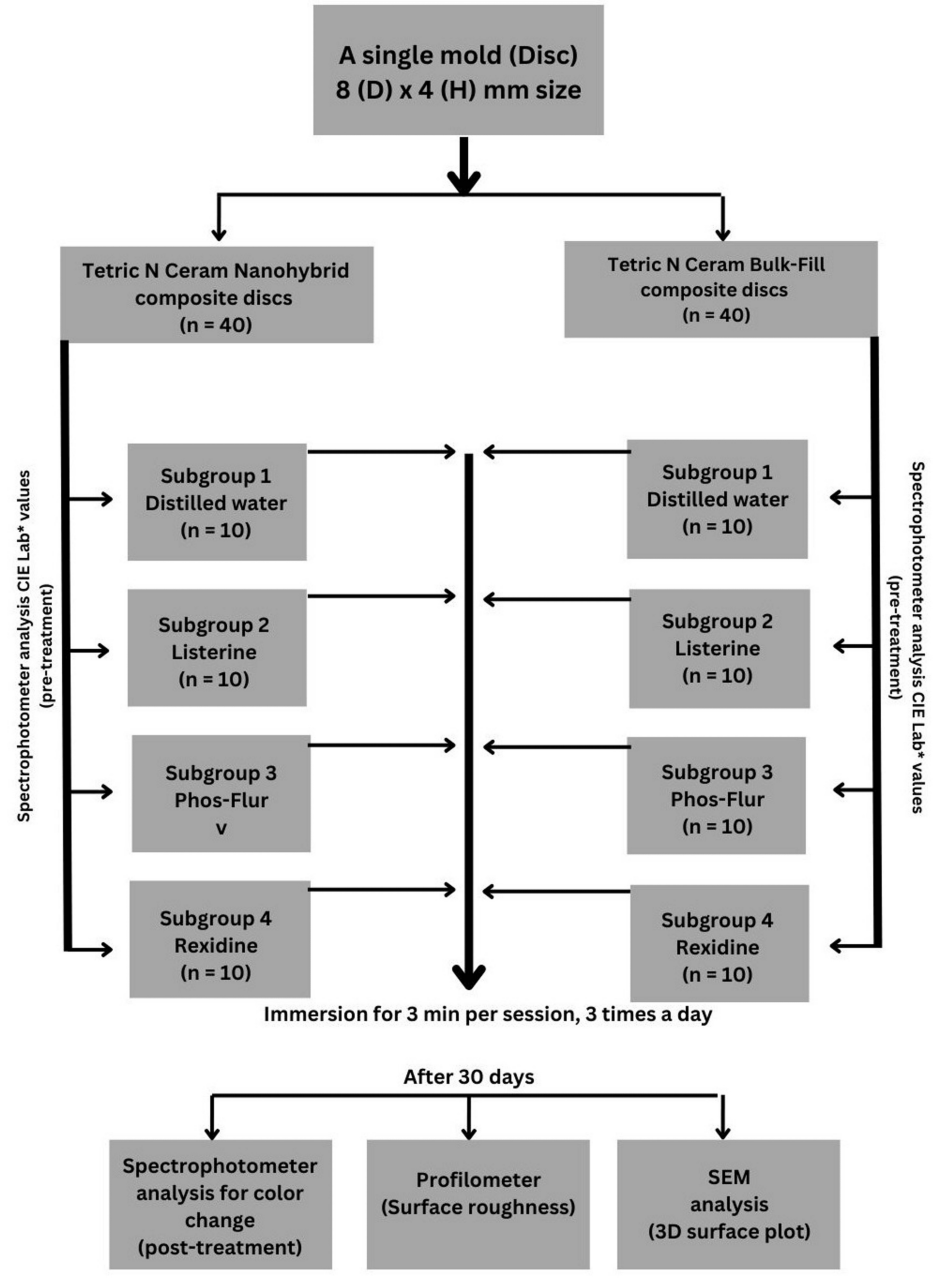
Study design CIE: Commission Internationale de l’Eclairage; D: Diameter; H: Height; SEM: Scanning electron microscopy; 3D: Three-dimensional

To accurately simulate one year of clinical mouthrinse use, the immersion protocol was adjusted based on typical patient usage patterns with approximately two rinses per day for one minute each, totalling approximately 730 minutes (12.2 hours) of exposure annually. To replicate this in vitro experiment, each specimen was immersed in 30 mL of the respective mouthrinse for five minutes per session three times daily over a period of 60 days. This resulted in a cumulative exposure time of 900 minutes (15 hours), which slightly exceeded the estimated annual exposure to provide a safety margin and ensure a comprehensive simulation of the long-term oral conditions (Figure 1). This protocol offers a time-compressed yet realistic approximation of the cumulative effects of daily mouthrinse use over one-year as suggested in a systematic review by Morais Sampaio et al. [15].

Thermocycling

To simulate the thermal stresses experienced in the oral cavity, all the specimens were subjected to thermocycling using a thermocycler (Atico Export, India). The process involved alternating immersion in water baths at 5°C and 55°C with a dwell time of 30 seconds in each bath and a 10-second transfer interval. The temperature was controlled within a range of ±2°C to ensure consistent thermal stress across all the samples.

Color-stability analysis

Color changes were measured using a VITA Easyshade V spectrophotometer (VITA Zahnfabrik, Bad Säckingen, Germany). The specimens were placed on a white background to minimize the influence of ambient lighting. Initial color readings were recorded according to the Commission Internationale de l’Eclairage (CIE) color system, with the probe positioned perpendicular to the specimen surface to ensure optimal contact without pressure. After 60 days of immersion, the specimens were rinsed, air-dried at room temperature for 24 hours, and re-measured under the same standardized conditions. The total color change (ΔE) was calculated using the formula: ΔE = √[(ΔL)² + (Δa)² + (Δb)²], where ΔL, Δa, and Δb represent the differences in the lightness, red-green, and yellow-blue axes, respectively.

Surface roughness assessment

The surface roughness (Ra) values were measured using a contact profilometer (Zeiss, Oberkochen, Germany) over a 4 mm trace length. Five readings were taken at random points on the surface of each specimen, and the mean Ra value was calculated to represent the surface roughness of each sample.

Surface texture analysis with SEM and ImageJ

**Figure 2:**
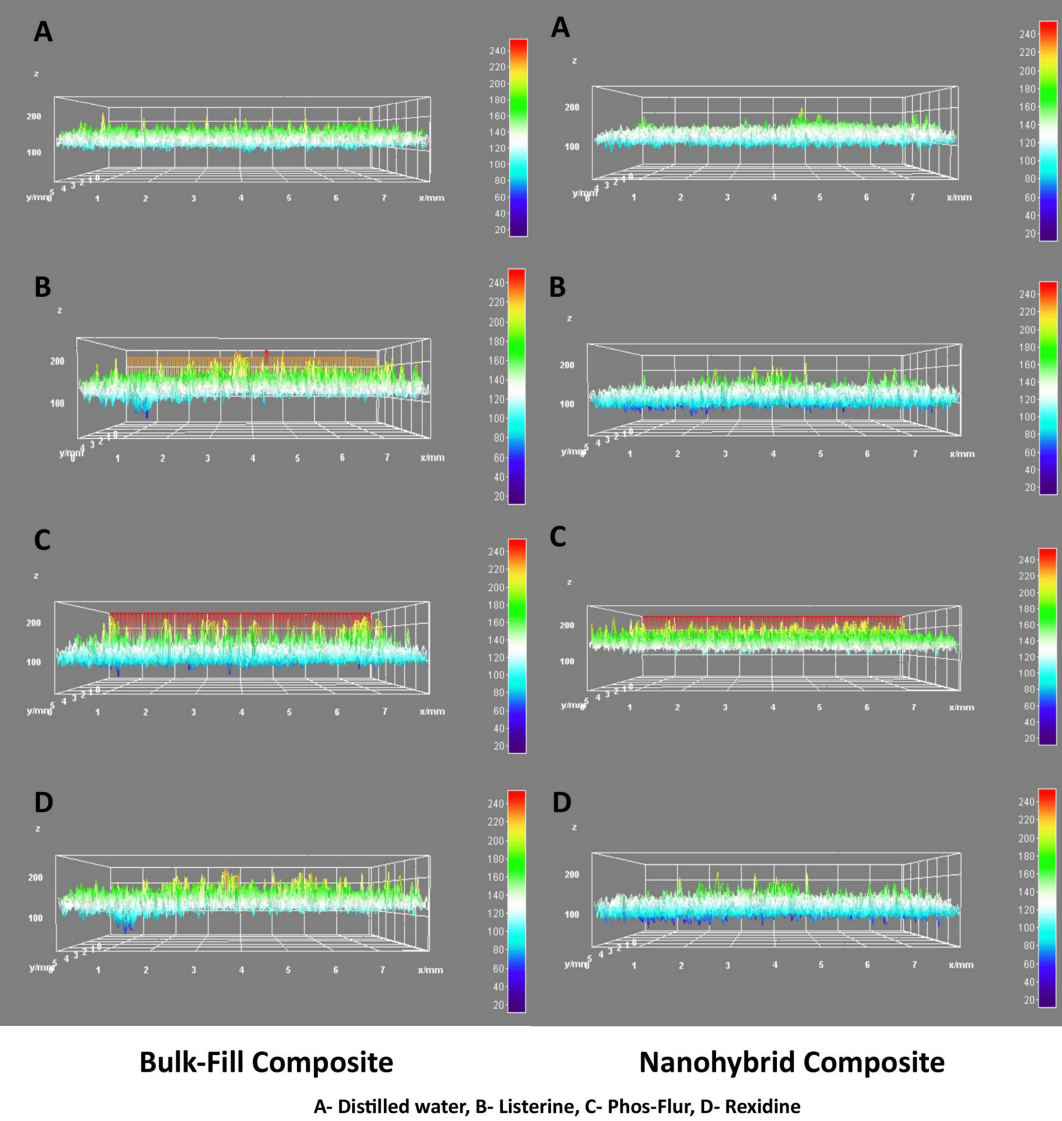
3D Surface plot of SEM images for analysis of surface roughness by immersion in different mouthrinses: (A) Distilled water; (B) Listerine; (C) Phos-Flur; and (D) Rexidine X axis denotes micrometer, Y axis denotes micrometer, Z axis denotes 0-200 color grading. This image was derived from the data of our study. SEM: Scanning electron microscopy

SEM images of each specimen were obtained at a magnification of 1000x (Zeiss, Oberkochen, Germany). The images were then analyzed using ImageJ software (National Institutes of Health, Bethesda, Maryland, USA) equipped with the SurfaceJ plugin to generate 3D surface plots. These plots were used to evaluate textural changes such as surface uniformity, height deviations, and topographical irregularities, as a complement to the profilometry results (Figure 2).

All outcomes were evaluated at the initial point of assessment and after a period of 60 days of exposure to mouthwash.

Calibration and reliability

To ensure consistency and minimize procedural bias, all specimen preparation, polishing, and finishing procedures were performed by a single experienced and calibrated operator (Shivani Bansal). Calibration was performed prior to the experimental phase by preparing a pilot batch of specimens and reviewing them for consistency in dimensions, surface quality, and curing uniformity. Instrumental calibration was performed according to the manufacturer’s guidelines. The VITA Easyshade® V spectrophotometer was calibrated before each use using a standard white reference block to ensure the accuracy of color measurements. Similarly, the profilometer was calibrated using a reference standard surface with known roughness values prior to specimen evaluation. Profilometric analysis was performed by a single trained examiner from a different college who was unaware of the group allocation (Saurabh Gupta). Similarly, for surface texture analysis, all SEM images were processed using the ImageJ software by a single trained and blinded examiner (Saru Gupta). To assess intra-examiner reliability, 10% of the SEM images were randomly re-analyzed after a one-week interval. The consistency of surface texture measurements from the 3D surface plots was evaluated using the intraclass correlation coefficient (ICC), with values above 0.80 considered acceptable.

Blinding

To achieve blinding in this study, specimens were assigned unique alphanumeric codes by a third person, concealing their group (Tetric N-Ceram Nanohybrid, Tetric N-Ceram Bulk-Fill, or immersion subgroups: control, Listerine, Phos-Flur, Rexidine) from the operator, examiners, and data analysts. A single, calibrated operator, unaware of group assignments, prepared, polished, and handled specimens, which were stored in identical, coded containers to prevent identification during immersion or thermocycling. Outcome assessments (color stability, surface roughness, SEM analysis) were conducted in a randomized order with coded labels, ensuring the operator and examiners remained blinded. Data analysts received datasets with coded labels, and group information was revealed only after statistical analysis was completed, minimizing bias and enhancing the study’s objectivity and reliability.

Statistical analysis

All statistical analyses were conducted using SPSS software (version 26.0; IBM Corp., Armonk, USA) by a statistician (Vishakha Grover) who was provided with coded data . Data normality was verified using the Shapiro-Wilk test, which was found to be normally distributed. Paired t-tests were used to compare pre- and post-immersion color changes (ΔE) and surface roughness (Ra). Intragroup comparisons for color change and surface roughness were performed using one-way analysis of variance (ANOVA), followed by post-hoc analysis using Tukey's test. Additionally, 3D surface plot analysis for the SEM image parameters was descriptively analyzed and correlated with the roughness data using Pearson’s correlation coefficient. Statistical significance was set at p < 0.05, and 95% confidence intervals were reported for all statistical comparisons.

## Results

Good reliability was demonstrated by an ICC value of 0.86. The results in table 1 indicated the intergroup comparison of the mean color change (ΔE) and surface roughness (Ra) between the bulk-fill and nanohybrid composites under different conditions. In the control group, both materials showed minimal color changes and surface roughness with no significant differences. Listerine exposure led to the highest color changes in both groups, especially bulk-fill (ΔE = 3.32). However, the difference between the materials was not statistically significant (p = 0.143). Phos-Flur exposure caused moderate color change (ΔE = 1.57) and surface roughness (Ra = 1.60) in both composites, with no significant intergroup differences. Notably, Rexidine caused a significantly greater color change in bulk-fill (ΔE = 2.78) than in nanohybrid composites (ΔE = 1.76), with a p-value of 0.007, indicating a statistically significant difference. However, the surface roughness values remained low and non-significant across all the subgroups. Although both groups showed color changes after immersion in mouthwashes, such variation was considered acceptable for all groups (ΔE ≤ 2.7), except for the bulk-fill composite in Rexidine (ΔE = 2.78) and Listerine (ΔE = 3.32) [16].

**Table 1:** Intergroup comparison of ΔE and Ra using independent t-tests *p-value < 0.05: significant Data is presented in form of mean and SD. ΔE: Color change; Ra: Surface roughness

Subgroups	Groups	ΔE		t-value	p-value	Ra		t-value	p-value
		Mean	SD			Mean	SD		
Control	Bulk-Fill	0.54	0.19	0.06	0.474	0.15	0.44	0.13	0.449
	Nanohybrid	0.52	0.35			0.10	0.90		
Listerine	Bulk-Fill	3.32	1.55	1.19	0.143	1.20	0.29	0.43	0.335
	Nanohybrid	2.58	0.13			1.11	0.64		
Phos-Flur	Bulk-Fill	1.57	0.75	0.01	0.495	1.60	1.10	0.02	0.499
	Nanohybrid	1.57	0.72			1.60	0.73		
Rexidine	Bulk-Fill	2.78	0.40	3.41	0.007*	0.18	0.35	0.04	0.481
	Nanohybrid	1.76	0.86			0.19	0.26		

One-way ANOVA analysis (Table 2) was used to evaluate color changes in the bulk-fill and nanohybrid composite groups exposed to different mouthrinses. Both groups showed significant color changes (p = 0.0001). These results suggest that the type of mouthwash significantly affected the color stability in both bulk-fill and nanohybrid composites, with distinct effects observed within each group.

**Table 2:** Comparison of color change for different mouthrinses within the group by one-way ANOVA test *p-value < 0.05: significant ANOVA: Analysis of variance

Groups	Sum of square	df	Variance	F-value	p-value
Bulk-Fill	47.7848	3	15.9283	20.15	0.0001*
Nanohybrid	21.587	3	7.1957	20.81	0.0001*

The post-hoc Tukey analysis revealed that Listerine caused a significantly greater color change compared to all other agents in both the bulk-fill and nanohybrid groups. Rexidine also showed a strong discoloration effect, particularly when compared with the control, but the difference was not statistically significant for bulk-fill. Phos-Flur generally resulted in the least color change among the agents, showing only borderline or non-significant differences when compared with Rexidine. Overall, the analysis highlighted that both Listerine and Rexidine contributed notably to discoloration, with more pronounced effects in bulk-fill than in the nanohybrid, emphasizing the differential susceptibility of the materials to staining agents (Table 3).

**Table 3:** Post-hoc Tukey analysis for pairwise comparison within the groups *p-value < 0.05: significant

Pairwise comparison		Bulk-Fill		Nanohybrid	
		Mean Difference	p-value	Mean Difference	p-value
Control	Listerine	2.78	0.0001*	2.06	0.0001*
	Phos-Flur	1.03	0.0502	0.99	0.0029*
	Rexidine	2.24	0.0001*	1.24	0.0002*
Listerine	Phos-Flur	1.74	0.0005*	1.06	0.0015*
	Rexidine	0.53	0.5330	0.82	0.0179*
Phos-Flur	Rexidine	1.21	0.0216*	0.24	0.7892

One-way ANOVA showed a statistically significant difference in the surface roughness between the groups (Table 4).

**Table 4:** Comparison of Ra for different mouthrinses within the group by one-way ANOVA test *p-value < 0.05: significant Ra: Surface roughness; ANOVA: Analysis of variance

Groups	Sum of square	df	Variance	F-value	p-value
Bulk-Fill	16.057	3	5.352	13.3054	0.0001*
Nanohybrid	15.882	3	5.294	11.6672	0.0001*

The post-hoc analysis for surface roughness indicated that the bulk-fill material exhibited significant differences between most solution comparisons, especially showing increased roughness with Phos-Flur and Listerine. However, Rexidine had a minimal effect on bulk-fill, with differences not reaching significance in several comparisons. In contrast, the nanohybrid showed more uniform sensitivity across agents, with significant roughness differences observed between the control and all other solutions. The results suggested that the nanohybrids were more prone to surface alterations, whereas the bulk-fill responded more variably depending on the specific agent (Table 5).

**Table 5:** Post-hoc Tukey analysis for pairwise comparison surface roughness within the groups *p-value < 0.05: significant

Pairwise comparison		Bulk-Fill		Nanohybrid	
		Mean Difference	p-value	Mean Difference	p-value
Control	Listerine	1.05	0.0038*	2.06	0.0098*
	Phos-Flur	1.45	0.0001*	0.99	0.0001*
	Rexidine	0.03	0.9996	1.24	0.9906
Listerine	Phos-Flur	0.40	0.5014	1.06	0.3770
	Rexidine	1.02	0.0051*	0.82	0.0211*
Phos-Flur	Rexidine	1.42	0.0001*	0.24	0.0002*

SEM 3D surface analysis revealed notable differences in the surface topographies of the two materials under certain conditions. Significant intergroup differences in the mean gray values were observed for the control and Listerine subgroups, suggesting distinct surface characteristics between the nanohybrid and bulk-fill when untreated or exposed to Listerine. However, the differences diminished under Phos-Flur and Rexidine exposure, with no significant variation, indicating that these agents had a comparable impact on the surface textures of both the materials. Overall, the findings suggested material-specific responses in the surface morphology depending on the type of exposure (Table 6).

**Table 6:** Intergroup comparison of mean gray value of SEM 3D surface plot analysis using independent t-tests *p-value < 0.05: significant Data is presented in form of mean and SD. SEM: Scanning electron microscopy; 3D: Three-dimensional

Subgroups	Groups	Mean grey value (SEM)		Minimum	Maximum	t-stat	p-value
		Mean	SD				
Control	Nanohybrid	92	18.4	58	150	4.42	0.003*
	Bulk-Fill	128	18.0	62	152		
Listerine	Nanohybrid	136	17.6	96	184	2.49	0.022*
	Bulk-Fill	152	10.0	118	168		
Phos-Flur	Nanohybrid	161	11.8	125	184	2.03	0.057
	Bulk-Fill	172	12.4	130	192		
Rexidine	Nanohybrid	138	16.6	86	169	0.51	0.613
	Bulk-Fill	134	18.2	77	168		

Table 7 showed the correlations between surface roughness (profilometer) and 3D surface plot (SEM) for bulk-fill and nanohybrid materials across the subgroups. For bulk-fill, correlations ranged from 0.65 (Listerine, p = 0.003) to 0.82 (Rexidine, p = 0.031), and for nanohybrid, from 0.72 (Listerine, p = 0.001) to 0.86 (Rexidine, p = 0.023). All correlations were significant (p < 0.05), this indicated a strong positive relationship between the profilometer and SEM measurements, with Rexidine exposure yielding the highest correlation.

**Table 7:** Correlation of Ra with profilometer and 3D surface plot of SEM images Pearson correlation test (r), p < 0.05 significant 0.6≤∣r∣<0.8: Strong; 0.8≤∣r∣≤1: Very strong Ra: Surface roughness; 3D: Three-dimensional; SEM: Scanning electron microscopy

Subgroups	Bulk-Fill		Nanohybrid	
	r value	p-value	r value	p-value
Distilled water	0.78	0.001*	0.82	0.002*
Listerine	0.65	0.003*	0.72	0.001*
Phos-Flur	0.75	0.027*	0.79	0.036*
Rexidine	0.82	0.031*	0.86	0.023*

## Discussion

This in vitro study aimed to evaluate and compare the effects of commonly used mouthrinses on the color stability, surface roughness, and surface morphology of two widely used composite resins, Tetric N-Ceram Nanohybrid and Tetric N-Ceram Bulk-Fill. These findings underscore the importance of understanding how different restorative materials respond to routine oral hygiene practices, particularly in relation to chemical exposure from mouthrinses.

Color stability is a critical property for the aesthetic success of composite restorations. In the present study, both composite types exhibited varying degrees of discoloration when exposed to different mouthrinses, consistent with previous literature that emphasizes the susceptibility of resin-based composites to staining agents over time [17,18]. Among the tested solutions, Listerine caused the most pronounced color changes, particularly in bulk-fill composites, followed by Rexidine mouthwash. This observation is likely attributed to the alcohol content in Listerine, which can alter the surface structure of the resin matrix, increase its permeability, and facilitate deeper pigment penetration. Ethanol, classified as a bipolar molecule, induces solubilization of both hydrophobic and hydrophilic constituents present within the resins. Research has indicated that dimethacrylates exhibit heightened vulnerability to the influence of alcohol [19]. A previous study by Leal et al. reported that bulk-fill composites showed high water sorption, leading to softening and matrix degradation of the composite resin and staining with Listerine [20]. In contrast, nanohybrid composites showed acceptable color change in all the mouthrinses, which might be due to the fact that they contain smaller, uniformly distributed nanosized filler particles and a higher filler loading, resulting in a smoother, more polishable surface that resists stain accumulation [13]. Additionally, they incorporated a more cross-linked and stable resin matrix, which minimized water sorption and pigment penetration. In contrast, bulk-fill composites often have a more flexible resin matrix and larger filler particles to facilitate bulk placement and curing; however, these features may compromise their resistance to discoloration [9]. Tetric N-Ceram Bulk-Fill, a nanohybrid composite from Ivoclar Vivadent, has a filler composition of approximately 77-79 wt.% (53-55 vol.%) inorganic fillers, including barium aluminum silicate glass, ytterbium trifluoride, and spherical mixed oxides, with particle sizes ranging from 40 nm to 3000 nm. The resin matrix, comprising 20-21 wt.% dimethacrylates (Bis-GMA, UDMA), includes a patented light initiator (Ivocerin®) for enhanced depth of cure, allowing bulk placement up to 4 mm [9]. Collectively, these compositional advantages make nanohybrid composites more color-stable when exposed to staining agents such as mouthwashes [21]. A systematic review by Morais et al. concluded that most mouthwashes did not cause any clinically acceptable color changes in the composite resins [15].

Phos-Flur, a fluoride-based mouthrinse, showed the least discoloration effect on both the composite materials. Its lower impact on color change may be linked to the absence of alcohol and relatively neutral pH, which minimizes resin matrix degradation [19]. Although Phos-Flur induced some changes in the color parameters, these were less significant and fell within the clinically acceptable range [16]. Rexidine, a chlorhexidine-based mouthrinse, also caused noticeable discoloration in bulk-fill composites, which might be due to the strong affinity of chlorhexidine for proteinaceous and resin-based surfaces, as noted by Furtado et al. [22]. Chlorhexidine causes extrinsic staining on composite restorations due to its cationic nature, which allows it to bind to negatively charged surfaces like composite resins. This binding can attract dietary chromogens (such as, from tea, coffee) or form complexes with salivary proteins, leading to stain accumulation. The stains typically appear as brown, yellow, or orange discoloration on the composite surface, especially with prolonged exposure, making this finding particularly relevant for patients using it for long-term antimicrobial therapy [13]. In a study by Valizadeh Haghi et al., it was found that chlorhexidine and alcohol-containing fluoride mouthwash led to less discoloration than Listerine mouthrinse, which could have been due to high alcohol content and low pH of Listerine [18].

Post-hoc analysis confirmed that Listerine and Rexidine caused significantly more color changes than the control and Phos-Flur subgroups. Interestingly, while Rexidine showed strong staining potential, it did not statistically differ from Listerine in the bulk-fill group, indicating that both agents possessed comparable discoloration potential under specific conditions. These results support the hypothesis that the chemical composition of mouthrinses, including their alcohol content, pH, and active agents, plays a crucial role in influencing the aesthetic durability of restorative materials [18].

Surface roughness is another critical factor, as it affects not only the visual quality of restorations but also their biofilm accumulation and long-term performance. In this study, Listerine and Phos-Flur significantly increased the surface roughness of both composite materials, although their effects were more pronounced in nanohybrid composites. Increased surface roughness may result from chemical degradation or leaching of the resin matrix and filler particles due to the acidic or alcohol-based nature of mouthwashes [10]. The nanohybrid composite, despite its advanced filler technology, appeared more sensitive to surface changes, possibly owing to its higher filler load and more complex particle distribution, which could expose the matrix to greater degradation during immersion [13]. In a study by Ayatollahi et al., neither alcohol nor non-alcohol mouthrinses led to significant surface roughness of the bulk-fill composites [23]. In contrast, Urbano et al. reported lower surface roughness of nanocomposites in an alcohol-based mouthrinse [24]. This could be due to the fact that the authors used Filtek Z350 nanocomposite, which showed lower surface roughness due to its nanocluster filler technology, which offers superior polish retention and smoother surface integrity. While Tetric N-Ceram contains a blend of nano- and micro-fillers, its larger particle size and mixed filler morphology make it more susceptible to surface degradation when exposed to chemical agents [21]. Tetric N-Ceram contains a filler composition of approximately 80-81 wt.% (55-57 vol.%) inorganic fillers, including barium glass, ytterbium trifluoride, mixed oxides, and copolymers, with particle sizes ranging from 40 nm to 3,000 nm. The fillers are combined with 19-20 wt.% dimethacrylates (resin matrix), and less than 1 wt.% additives, initiators, stabilizers, and nano-color pigments, which contribute to its chameleon effect, radiopacity, and low shrinkage. This nano-optimized filler technology enhances mechanical properties, polishability, and esthetics [21]. Additionally, tighter filler-matrix integration in Filtek Z350 provided better resistance to erosion and filler dislodgement, contributing to its smoother post-immersion surface profile.

Interestingly, Rexidine had a minimal effect on the surface roughness of the bulk-fill, indicating that the resin formulation or filler content in bulk-fill composites may provide better resistance against chlorhexidine-induced surface alteration [7]. Conversely, the nanohybrid composites showed significant changes even with Rexidine, underscoring the variability in the material behavior. These findings are clinically significant because surface roughness above a certain threshold (typically Ra > 0.2 µm) is associated with increased plaque retention and staining, potentially compromising the longevity and appearance of restorations [25].

SEM and 3D surface plot analyses further complemented the roughness data by revealing the topographical changes induced by different mouthwashes. The mean gray value analysis suggested that Listerine caused the most distinct surface alterations. The pronounced changes in the nanohybrid under Listerine exposure likely reflect the softening or erosion of the surface matrix, leading to greater light absorption and scattering on SEM imaging [10,19]. The less pronounced differences between the Phos-Flur and Rexidine groups suggest a more uniform effect on both composites, likely due to the milder nature of these solutions or their reduced interaction with the filler particles [19].

These surface texture results are essential for understanding how microscopic changes translate into macroscopic effects, such as roughness and discoloration. A higher mean gray value indicates smoother or more reflective surfaces, whereas a lower value may indicate increased surface irregularities. The alignment of the SEM findings with the profilometric data supports the reliability of the results and strengthens the conclusions of this study.

The robustness of this study is attributable to the implementation of a standardized methodology. All specimens were meticulously prepared, polished, and assessed under controlled laboratory conditions, to minimize variability. The use of calibrated instruments and a single operator for all procedures ensured consistency across samples. The high ICC value (0.86) for intra-examiner reliability further supports the validity of the findings. Additionally, simulating oral conditions using thermocycling and immersion protocols adds clinical relevance by mimicking the thermal and chemical challenges faced by restorations in vivo.

Despite these strengths, this study has several limitations. As an in vitro study, the findings might not entirely replicate clinical conditions, where factors such as salivary enzymes, mechanical wear from mastication, and oral hygiene practices may further influence material behavior. Moreover, only one brand and formulation of each composite type were tested. The results might vary for different formulations, particularly those with alternative resin matrices or filler technologies.

Clinically, the results of this study suggested that restorative material selection should consider the patient’s oral hygiene regimen. For patients using alcohol-based or chlorhexidine mouthrinses, especially over prolonged periods, nanohybrid composites are more vulnerable to color instability and surface degradation. Practitioners might consider recommending non-alcoholic or neutral-pH mouthwashes (such as RiseWell Balancing Mouthrinse, CloSYS Sensitive Mouthrinse, ORL Natural Mouthrinse) for such patients or opting for restorative materials with higher resistance to these effects.

## Conclusions

The study showed that mouthrinses with varying alcohol content, and chemical compositions caused differential staining, surface degradation, and roughness on composite resins by interacting with their resin matrix and filler particles. Listerine and Rexidine caused the most substantial color changes, particularly in the bulk-fill composite. The nanohybrid composite consistently exhibited better color stability. While the surface roughness remained low across all groups, the nanohybrid showed greater sensitivity to surface alterations across different solutions, whereas the bulk-fill displayed variable responses in surface roughness, with significant increase under Phos-Flur and Listerine but minimal impact from Rexidine, unlike the nanohybrid's more uniform sensitivity across all solutions. SEM analysis further confirmed these findings, revealing distinct surface changes between the two materials, especially under Listerine exposure. Overall, the results highlighted the differential behavior of composite resins when exposed to common mouthrinses, emphasizing the importance of material selection based on a patient’s oral hygiene practices to maintain long-term aesthetic outcomes. 
